# Mindfulness-based family psychoeducation intervention for caregivers of young adults with first-episode psychosis: results at 9-month follow-up

**DOI:** 10.3389/fpsyt.2024.1460151

**Published:** 2024-09-26

**Authors:** Zoe Jiwen Zhang, Herman Hay Ming Lo, Wing Chung Ho, Elsa Ngar Sze Lau, Siu Man Ng, Winnie W. S. Mak, Samuel Yeung Shan Wong, Karen S. Y. Hung, Iris Yuen Shan Lai, Cola Siu Lin Lo, Jessica Oi Yin Wong, Simon S. Y. Lui, Clara Man Wah Siu, Eric Wai Ching Yan, Sunny Ho Wan Chan, Edmund Lin, Gloria Oi Chi Wong, Jonathan Wai Hung Mak, Hillman Shiu Wah Tam, Iris Huen Hung Tse

**Affiliations:** ^1^ Department of Applied Social Sciences, Hong Kong Polytechnic University, Hong Kong, Hong Kong SAR, China; ^2^ Department of Social and Behavioural Sciences, City University of Hong Kong, Hong Kong, Hong Kong SAR, China; ^3^ Department of Educational Administration & Policy, Chinese University of Hong Kong, Hong Kong, Hong Kong SAR, China; ^4^ Department of Social Work and Social Administration, University of Hong Kong, Hong Kong, Hong Kong SAR, China; ^5^ Department of General Adult Psychiatry, Castle Peak Hospital, Hong Kong, Hong Kong SAR, China; ^6^ Kowloon Hospital, Hospital Authority, Hong Kong, Hong Kong SAR, China; ^7^ Centre for Health and Clinical Research, University of the West of England, Bristol, United Kingdom; ^8^ Lingnan University, Hong Kong, Hong Kong SAR, China; ^9^ Hong Kong Family Welfare Society, Hong Kong, Hong Kong SAR, China; ^10^ Heartfelt Listening Counselling Space, Hong Kong, Hong Kong SAR, China

**Keywords:** mindfulness-based program, caregivers, first-episode psychosis, positive caregiving experience, expressed emotions

## Abstract

**Objectives:**

To investigate the effects of a mindfulness-based family psychoeducation (MBFPE) intervention on caregivers and the young adults with first-episode psychosis in mental health care.

**Methods:**

Sixty-five caregivers were randomly assigned to the MBFPE program (n = 33) or an ordinary family psychoeducation (FPE) program (n = 32). Eighteen young adults in recovery (YAIR) also participated in the study. All of the participants completed the assessments before participating in the intervention (T1), after the intervention (T2), and at 9-month follow-up (T3).

**Results:**

Intention-to-treat analyses were conducted. The caregivers reported a significant and large effect size on positive caregiving experiences based on a Time × Group analysis (*g* = 0.862, *p* = 0.006). Among the YAIR participants, between-group differences were significant in their perceptions of caregivers’ expressed emotions, including large effect sizes of perceived criticism (*g* = 1.396, *p* = 0.049) and hostility (*g* = 1.444, *p* = 0.043). Caregiver demographics, including age, education level, socioeconomic status, and number of family members, were found to moderate the effect sizes of the variables studied.

**Conclusion:**

This study provides evidence of the effects of MBFPE programs on the outcomes of caregivers and the young adults with first-episode psychosis in their care. Specifically, the MBFPE program in this study played a greater role in promoting positive caregiving experiences and changing caregivers’ expressed emotions, especially their expressed criticism of YAIR, compared with the regular FPE program. Therefore, the application of mindfulness training to promote family care and YAIR recovery should be encouraged.

**Clinical trial registration:**

ClinicalTrials.gov, identifier NCT03688009.

## 1 Introduction

Psychosis refers to the group of severe mental disorders characterized by hallucination and delusions. Individuals with psychosis also suffer serious impairments in cognitions and social functioning (1). The prevalence of psychosis in general public is around 0.7 to 2.5%, while a marked increase has been found in the range from 15 to 17 years old, and the majority of suffering from psychosis is the population aged 20 to 30 years old (2, 3).

Psychosis can be a long-term illness (in particular for those with schizophrenia), posing lifelong challenges to patients. Research has revealed that the five-year relapse rate can be as high as 80% among individuals suffering from schizophrenia, and the rate of suicidality of this population is up to 10% (4). Alongside the psychiatric symptoms, young adults with psychosis are particular vulnerable to developing negative outcomes, such as the stigma of living with psychosis, impairments of social functioning, and difficulties in social integration. To address these challenges, it is essential for them to gain interactions, support, and understanding from caregivers (5).

A high level of caregiver burden has been reported by caregivers of family members with mental illness, which seriously affects their own mental health (6). Caring for family members with psychosis requires endless energy and empathy from caregivers, who must exert great effort to balance their professional and family roles. However, caregivers’ efforts may not receive adequate recognition or financial support in most countries (7). Glecia and Li (8) showed that caregivers’ mental health and well-being were affected by increased burden, high levels of emotional and physical stress, and poor quality of life due to restricted social life, safety concerns, and a lack of formal and informal support.

Marked negative responses have been reported by caregivers of people with psychosis, such as depression, anxiety, guilt, self-blame, and somatic complaints (9, 10). Expressed emotions (EEs), which refer to the emotional characteristics expressed by caregivers toward their family members, have been increasingly studied from the perspective of family dynamics (11). EEs include three domains, namely criticism, hostility, and over-involvement (12). Studies have shown that EEs are a significant predictor of psychosis relapse (12, 13) and that the different EE domains have detrimental impacts on increasing positive symptoms and decreasing quality of life in people with psychosis (14, 15).

Studies have documented the role of EEs, showing their negative impacts on psychosis relapse in different cultural contexts (16, 17). From the perspective of family dynamics, over-involvement has different meanings and signs in a family with strong ties, which is commonly seen in Asian and other collective cultures. Recent critiques of the concept of EE have suggested that over-involvement can be seen as an attempt by family members to actively participate in care and should not be considered a sign of family dysfunction (16, 18). Although criticism generally brings negative impact to most family members, in the context of family caregiving, it may be interpreted by patients as a sign of warmth, indicating that caregivers are urging young adults in recovery (YAIR) to improve or recover from their psychosis (16, 19, 20). Thus, striking a balance in attaining optimal level of family involvement for each person with psychosis is critical in affecting personal recovery (21). The role of EEs in caregivers’ mental health and the recovery of young adults with psychosis warrants further research in different cultural contexts.

Family psychoeducation (FPE) programs have been shown to improve the course of psychosis (22, 23). Common needs of caregivers include emotional support, recognition of their caregiving role and contributions, relief from psychosis-related social isolation, and reliable services (24). FPE programs generally use the cognitive behavioral approach to support caregivers and teach them practical skills, which can solve some of their problems and help them access resources quickly and inexpensively (24). The results of a meta-analysis conducted by Falloon showed that the one-year relapse rate of FPE program participants was approximately 6% to 12%, while that of the participants in the control group was 41% to 53% (25). The meta-analysis conducted by Sin et al. further confirmed improvements in caregivers’ well-being, overall morbidity, perceived burden, negative caregiving experiences, and EEs in their selected 32 randomized controlled trials (RCTs) (26). However, the results for positive caregiving experiences, coping, family functioning, and perceived social support were not significant. Finally, a meta-analysis conducted by Yesufu-Udechuku et al. concluded that caregivers of people with serious mental illnesses reported improvements in their caregiving experience after psychoeducation programs, but the quality of the data was low and limited by small and heterogeneous samples (27).

Mindfulness-based programs (MBPs) have been developed and applied to improve the awareness and insight of individuals experiencing from chronic illness or living in an environment characterized by chronic illness (28). MBPs have shown promise in enhancing various aspects that can benefit caregivers, such as improving attention, fostering tolerance of unpleasant sensations and feelings, facilitating cognitive changes, and promoting effective coping strategies. These benefits can be instrumental in aiding caregivers in handling the challenges associated with caregiving burden and EE. Recent research indicates that mindfulness plays a crucial role in adaptive emotion regulation. MBPs have demonstrated their impact in reducing the intensity of emotional distress, enhancing emotional recovery, decreasing negative self-referential processing, and encouraging engagement in goal-directed behaviors (29). In the intricate dynamics of caregiving relationships, family caregivers often face stress related to monitoring psychotic symptoms and providing care to individuals with psychosis who may lack insight into their own care needs (30). Additionally, the impact of psychosis can extend to the entire family, disrupting the functioning of other members and leading caregivers to feel overwhelmed by anxieties and a sense of diminished abilities (31). Through mindfulness exercises such as mindful breathing, stretching, sitting, and body scanning, caregivers can regulate their emotions and use their curiosity and open-mindedness to become aware of what is happening and further improve their acceptance (28).

Mindfulness-based family psychoeducation (MBFPE) programs have been shown to be an important component of treatment aimed at promoting family care and the well-being and recovery of family members with chronic illness (32–34). In a study conducted specifically among parents of children with mixed psychiatric diagnoses or chronic illnesses, Boügels et al. showed that parents reported a reduction in depressive and anxiety symptoms. However, further studies are needed to investigate the effects of mindfulness and explore whether MBPs can improve caregivers’ negative mental health symptoms and positive psychological well-being (35).

We developed a brief MBFPE program for caregivers of young adults in recovery (YAIR) who experienced their first-episode of psychosis within the last three years. Only the caregivers participated in the MBFPE program, but we collected data from both caregivers and YAIR participants. We also assigned caregivers to an ordinary FPE program as an active control group and compared their outcomes before the program, after the program, and at 9-month follow-up. The results of the immediate effects after the completion of the MBFPE program were reported in a previous paper (36), and this paper focuses on the results at 9-month follow-up.

In this study on the effects of the MBFPE program, we investigated changes in both caregivers’ negative symptoms and their positive caregiving experiences. Lo et al. showed an increase in positive affect among people with recurrent depressive and anxiety symptoms (37), but parents of children with attention-deficit/hyperactivity disorder did not show improvements in psychological well-being after an MBP (38). It is possible that an MBP helps to improve caregivers’ awareness and insight, allows them to observe and appreciate YAIR’s recovery, and helps them to understand the full experience of caregiving. In our qualitative study of caregivers of young adults with psychosis, we applied the qualitative method called Photovoice (39). Using photos and sharing, caregivers expressed their positive experiences of caregiving, such as paying attention to the present moment, showing trust in their children, appreciating their connection with and support from nature and the universe, and finding space to care for others and themselves. However, such positive experiences did not appear in the quantitative data after the intervention (36). YAIR may have a different perspective from caregivers, so their point of view should be included (40). Jansen et al. further pointed out that higher levels of negative beliefs regarding uncontrollability and danger promote over-involvement, leading to increased distress (18). Metacognition contributes to distress by prompting people to adopt coping strategies. Although metacognition cannot reduce suffering and distress, which are common reactions when a family member suffers from psychosis, it helps develop an understanding of self and others and allows for a more balanced perspective on caregiving, including positive caregiving experiences.

In this study, the focus was on measuring the outcomes for caregivers who were in arm 1 (the MBFPE). This arm served as the experimental group in this study. The MBFPE program combined mindfulness exercises with psychoeducation with the goal of improving caregiver’s mental health and emotion regulation abilities, especially in reducing their expressed emotions while providing care to the young adults with psychosis. In contrast, arm 2 (FPE) served as the control group. It primarily concentrated on providing information and social support to the caregivers without the incorporation of mindfulness exercises. We investigated the effectiveness of the MBFPE program for caregivers and the YAIR in their care compared with the effectiveness of an ordinary FPE program. Considering the high heterogeneity of participants in previous studies, we also explored the moderating effects of caregivers’ basic information on the outcomes of the MBFPE program, including their demographic information, caregiving hours, and satisfaction with the program.

We examined the following three hypotheses:

Hypothesis 1: Caregivers in the MBFPE program show less caregiver burden, gain more positive caregiving experiences, have reduced physical distress, depression, and anxiety, and report higher levels of well-being, mindful parenting, and non-attachment across their pre-test, post-test, and 9-month follow-up, compared with those in the control group.

Hypothesis 2: The YAIR participants whose caregivers participated in the MBFPE program report higher levels of recovery, perceive lower levels of caregiver EEs, and lower levels of psychopathological symptoms across their pre-test, post-test, and 9-month follow-up, compared with those YAIR whose caregivers participated in the control group.

Hypothesis 3: Demographic information of caregivers, caregiving hours, and their satisfaction with the programs should moderate their changes in caregivers’ burden, caregiving experiences, physical distress, depression, anxiety, well-being status, mindful parenting, and non-attachment.

## 2 Methods

### 2.1 Study design

This study used a two-arm RCT to compare the effects of the MBFPE program (Arm 1) with those of the ordinary FPE program (Arm 2). The MBFPE and FPE programs included a 1-hour standardized psychoeducation video. Another hour involved mindfulness training in the MBFPE program and a sharing and discussion session in the FPE program. The participants were asked to complete the assessments before participating in the intervention (T1), after the intervention (T2), and at 9-month follow-up (T3).

### 2.2 Participants

We used convenience sampling due to the confidentiality of the YAIR participants’ medical records and their caregivers’ personal data. The sample size was calculated by using G*Power 3.1, with an effect size of 0.64 in depression, a two-tailed α error of 5%, and an 80% power (41). The sample size of caregivers was estimated to be 80 in total. The participants were recruited through two Early Assessment Service for Young People with Early Psychosis (EASY) clinics at Castle Peak Hospital and Kowloon Hospital and through a non-governmental organization (NGO) in Hong Kong. Research assistants helped the two EASY teams recruit participants from their outpatient units. Social workers at the NGO also referred caregivers who were interested in participating in the study, thus providing the largest number of caregivers for the study.

In terms of inclusion criteria, the participants had to be caregivers who had provided care to a YAIR for at least a year, and the YAIR participants had to have had their first episode of psychosis within the last three years, including schizophrenia spectrum, bipolar disorder, and other related psychotic disorders (Diagnostic and Statistical Manual of Mental Disorders, fifth edition [DSM-5]). In addition, the YAIR participants had to be able to provide informed consent and complete the assessments independently. In terms of exclusion criteria, all caregivers unable to independently understand the content of the programs were excluded, such as those with severe cognitive impairment or developmental disabilities. If the YAIR participants or their caregivers refused to receive regular psychiatric consultations, they were also excluded from the study.

**Figure 1:**
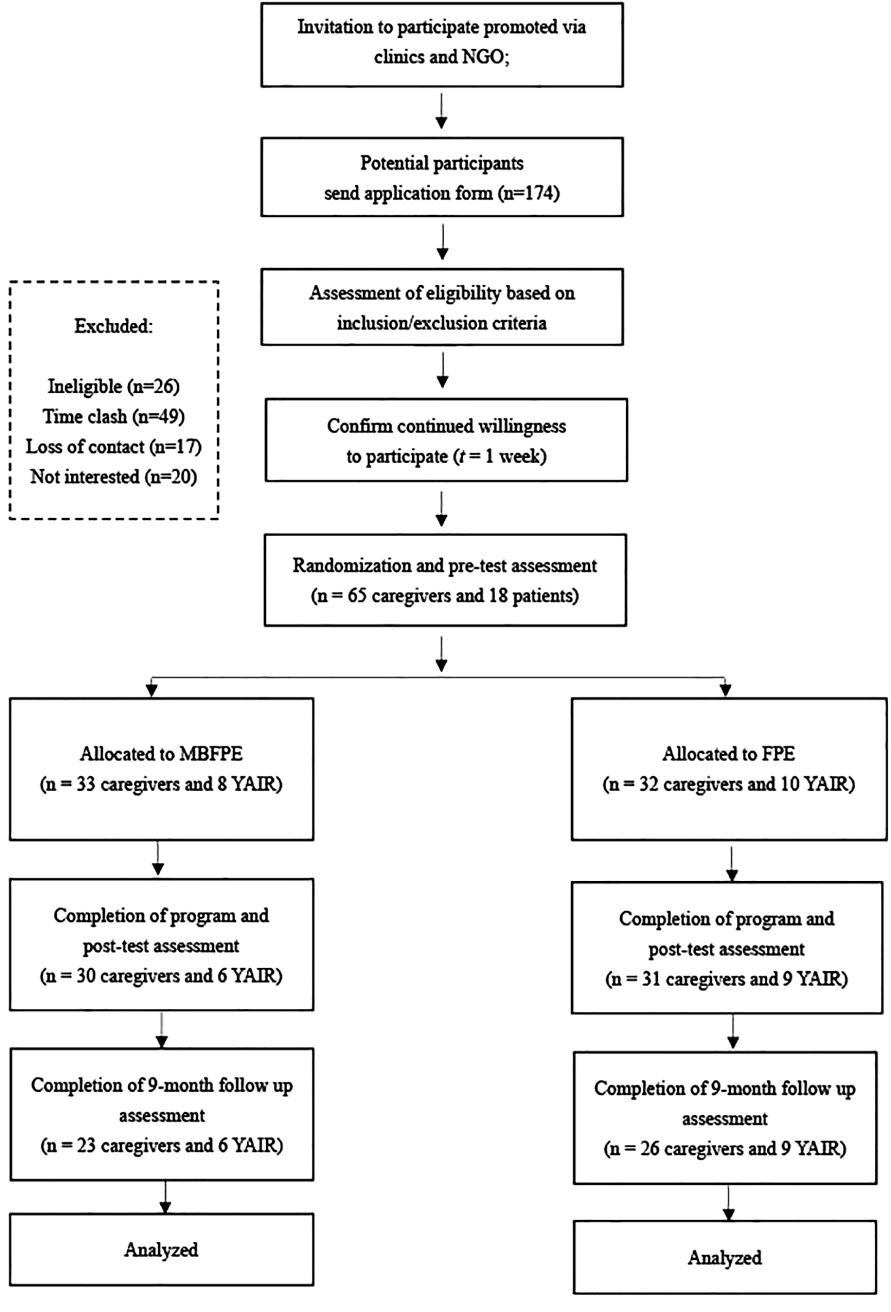
Flow chart of the process for randomized controlled trial.

Initially, 174 caregivers applied to participate in the study after they had received the program information from the psychiatrist or nursing officer at out-patient service, or the NGO newsletter. Some of them were excluded for the following reasons: loss of contact (n = 17), ineligibility for the study (n = 26), lack of interest during the recruitment process (n = 20), and time conflicts with the study arrangements (n = 46). Ultimately, 65 caregivers were included in the final sample. It was acknowledged that the number of caregivers was slightly lower than the initially estimated sample size. This decrease was primarily attributed to challenges in recruiting participants during the COVID-19 pandemic. The YAIR in their care were also invited to answer some of the research questions. Some of the YAIR did not want to spend time on the intervention program and were not interested in participating in the study (n = 47). Finally, 18 YAIR participants were included in the study. **Figure 1** shows the participant flowchart.

### 2.3 Procedures

The 65 eligible caregivers were randomly assigned to the MBFPE (arm 1) or FPE (arm 2) program by a computer program. They were told that they were participating in a “family psychoeducation program” without mentioning whether it contained mindfulness training. Both programs consisted of six weekly sessions each lasting 2 hours conducted face-to-face. The interventions in both arm 1 and arm 2 were conducted in group formats, consisting of 6 to 10 caregivers in each group. The intervention of this project was conducted from January 2019 to March 2021.

In arm 1, the MBFPE program included mindfulness exercises integrated with psychoeducation. Qualified instructors led the first hour of the MBFPE sessions engaging participants with included mindfulness exercises including body scanning, mindful stretching, mindful walking, mindful sitting, mindfulness for difficult times, and befriending (29, 42). Participants in arm 1 received 10-minute audio files after each session for daily mindfulness homework. During the second hour of MBFPE sessions, pre-recorded psychoeducation videos were utilized to cover topics including understanding symptoms of psychosis, medication, treatment management, collaboration among different mental health professionals, communication strategies, problem-solving techniques, and crisis management (43, 44). These videos were designed based on the expertise and best practices of multi-disciplinary mental health professionals, including psychiatrists, psychiatric nurses, clinical psychologists, occupational therapists, and social workers. In arm 2, the control group, the FPE program was conducted. Psychoeducation was integrated into sharing and discussion focused on providing emotional support among participants. The same set of psychoeducation videos were shown during the two-hour FPE session. Throughout both arms, instructors were responsible for organizing the video presentations, addressing questions relating to the psychoeducation content, and facilitating participant sharing and discussions. Regular breaks were scheduled every 15 to 20 minutes during the video sessions to engage the participants. In arm 1, the time allocated for sharing and discussion was roughly 15 minutes, which was influenced by the mindfulness content. In arm 2, the time dedicated to sharing and discussion extended to approximately 60 minutes.

In arm 1, the instructors were seasoned three mental health professionals holding master’s or doctoral degrees in social work, family therapy, or clinical psychology. They had completed the foundational professional training of mindfulness-based cognitive therapy at the Oxford Mindfulness Centre (now Oxford Mindfulness Foundation). These instructors had 5 to 20 years of experience in mental health care practice, with at least 3 years of teaching mindfulness-based programs. For arm 2, the three instructors were also experienced mental health professionals with master’s or doctoral degrees in social work, or clinical psychology, possessing expertise in mental health practice ranging from 3 to 15 years. Given the instructors’ extensive experience and expertise, supervision was deemed unnecessary. However, orientation and consultation sessions were conducted for individual instructors before they commenced teaching the program, and additional sessions were provided on need basis. The individual responsible for conducting the orientation and consultation sessions held a PhD and had completed professional training in mindfulness-based cognitive therapy and mindfulness-based stress reduction. With over 15 years of experience in teaching mindfulness-based programs, this individual ensured that the instructors were well-prepared and supported. All instructors delivered the program following intervention protocols developed by the corresponding author. The themes and session outlines can be found in **Appendix 1**, and the full protocol is available upon request by contacting the corresponding author.

After completing data collection at T2 and T3, the caregivers received a cash voucher worth HKD100 (approximately USD 12). The YAIR participants who completed data collection at the three time points also received a cash voucher worth HKD100 as an incentive to increase their engagement. All participants voluntarily participated in the study by giving their written content. They were also informed that the study was independent of their health care services and that they could withdraw at any time without negative responses or consequences. This study was part of a project registered with the United States Clinical Trials Registry (NCT03688009).

### 2.4 Measures

#### 2.4.1 Primary outcome variable

##### 2.4.1.1 Caregiver burden

This study used the Zarit Burden Interview (ZBI) to examine caregiver burden (45). This 22-item scale was used to measure caregivers’ health, psychological well-being, relationships with patients, social life, and finances (e.g., “Do you feel that you have lost control of your life since your relative’s illness?”). The items were rated on a 5-point Likert scale ranging from 0 (“not at all”) to 4 (“extremely”), with a higher score indicating a higher level of caregiver burden. We used the Chinese version of the ZBI, which was validated by Tang et al. for its good psychometric properties (Cronbach’s alpha = 0.88) and had high internal consistency (Cronbach’s alpha = 0.933) in this study (46).

#### 2.4.2 Secondary outcome variables

##### 2.4.2.1 Caregiving experiences

The Experience of Caregiving Inventory (ECI) was adopted to assess the caregiving experiences of the participating caregivers (47). We used three 5-point Likert subscales based on the objectives and content of this study, namely the Stigma, Effects on Family, and Positive Experience in Caregiving subscales. A total of 26 items were included. Sample items include “feeling unable to tell anyone about his illness” for stigma, “how family members do noy understand your situations” for effects on family, and “I have discovered strengths in myself” for positive caregiving experiences. The Chinese version of the ECI was validated by Lau and Pang, with Cronbach’s alpha values ranging from 0.75 to 0.85 (48). In this study, Cronbach’s alpha values were 0.766, 0.828, and 0.824 for the stigma, effects on family, and positive caregiving experience subscales, respectively.

##### 2.4.2.2 Caregivers’ physical health

We used the Physical Distress subscale of the Body–Mind–Spirit Well-Being Inventory (BMSWBI) to measure the physical health status of the participating caregivers, such as chest pain, headaches, and fatigue (49). The subscale included 14 items rated from 0 (“no distress at all”) to 10 (“extreme distress”). Cronbach’s alpha was 0.948 in this study.

##### 2.4.2.3 Caregivers’ mental health symptoms

We used the Hospital Anxiety and Depression Scale (HADS) to measure the mental health symptoms of the participating caregivers, consisting of 14 items, ranging from 0 (“low”) to 4 (“severe”), with a maximum score of 21 for anxiety or depression (50). Among these 14 items, seven items were used to measure depressive symptoms and seven to measure anxiety symptoms. In the current study, Cronbach’s alpha values were 0.745 and 0.851 for depression and anxiety, respectively.

##### 2.4.2.4 Caregivers’ well-being

We adopted the World Health Organization—Five Well-Being Index (WHO-5) to measure positive aspects of mental health among the participating caregivers (51). The WHO-5 consists of five items prompting caregivers to reflect on their well-being over the last two weeks. The items were rated from 0 (at none the time) to 5 (all of the time), with a higher score indicating better perceived well-being. Cronbach’s alpha was 0.947 in this study.

##### 2.4.2.5 Caregivers’ interpersonal mindfulness

The Interpersonal Mindfulness in Parenting Scale (IM-P) is a scale used by parents to assess their mindful parenting (52). Lo et al. validated the Chinese version of the IM-P, which includes 23 items with four subscales, namely compassion for children, emotional awareness in parenting, non-judgmental acceptance in parenting, and listening with full awareness (53). Cronbach’s alpha was 0.852 in this study, indicating good internal consistency.

##### 2.4.2.6 Caregivers’ non-attachment

We adopted the Non-Attachment Scale to measure the general state of the participating caregivers’ psychological and social adaptation (54). The Chinese version—Short form revised by Chio et al. was validated with eight items, ranging from 1 (“strongly disagree”) to 6 (“strongly agree”) (55). Cronbach’s alpha was 0.871 in this study, indicating the good psychometric properties of the scale.

##### 2.4.2.7 Young adults’ recovery level

The Mental Health Recovery Measure is a 5-point Likert scale designed to assess the level of mental health recovery in young adults (56). Rather than measuring young adults’ psychiatric symptoms, the scale contains 30 items related to their experience of psychosis. The measure includes multiple aspects, such as self-empowerment, spirituality, and overcoming “stuckness.” The measure had good internal consistency in this study (Cronbach’s alpha = 0.944), echoing the findings of Ye et al., who validated the Chinese version of the scale (57).

##### 2.4.2.8 Young adults’ family EEs

We adopted the Level of Expressed Emotion Scale (LEES), which is a 12-item self-reported questionnaire used to measure family EEs among the YAIR participants (13). This measure consists of three subscales: criticism, hostility, and over-involvement. Each item was rated from 0 (“totally disagree”) to 4 (“totally agree”), with a higher score indicating a higher level of criticism, hostility, or over-involvement. The general level of family EEs was the sum of the scores on the three subscales. In this study, the full LEES had good internal consistency (Cronbach’s alpha = 0.924), and the Cronbach’s alpha values were 0.744, 0.938, and 0.854 for the subscales respectively.

##### 2.4.2.9 Young adults’ psychiatric symptoms

We used the Positive and Negative Syndrome Scale (PANSS) to measure the YAIR participants’ psychiatric symptoms (58, 59). In this study, the PANSS was scored by the a psychiatrist or research assistants on the research team with at least three years of experience in mental health practice. The measure included seven items for positive symptoms (e.g., delusions), seven items for negative symptoms (e.g., emotional withdrawal), and 14 items for general psychopathology (e.g., somatic concerns), each ranging from 0 (“absent”) to 7 (“extreme”). The Chinese version was validated by Chan et al. with good internal consistency (60). In the current study, the values of Cronbach’s alpha for positive symptoms, negative symptoms, and general psychopathology were 0.767, 0.902, and 0.796, respectively.

##### 2.4.2.10 Dosage and participant satisfaction

Attendance and time spent in home practice were recorded as dosage. After completing the program, all participating caregivers were asked to rate their satisfaction with the program using a 4-point questionnaire (from 1 “very dissatisfied” to 4 “very satisfied”).

##### 2.4.2.11 Fidelity to the MBFPE program

To ensure fidelity to the MBFPE program, all sessions were audio recorded. We randomly selected 20% of the clips for independent evaluation to assess the quality and consistency of the programs. In addition, we adopted the Mindfulness-based Interventions: Teaching Assessment Criteria Scale (MBI: TAC) to verify fidelity to the MBFPE program (61).

### 2.5 Data analysis

Intention-to-treat analysis was used for data analysis and the multiple imputation method was used to deal with missing data (62, 63). The participants who completed 50% or more of the MBFPE or FPE sessions in their post-test and follow-up test were included in this study to evaluate the respective programs. In this trial, a repeated measurements approach was adopted to ensure that all collected data was utilized for analysis. Upon observation, it was noted that no systematic differences between the caregivers with complete data and those with missing data. This observation led to the assumption that the missing data was missing at random (MAR), prompting the need for effective missing data management (64). To address the missing data, multiple imputation was conducted according to the types of variables. Predictive mean matching (PMM) was employed for used for continuous variables, logistic regression was used for dummy variables, multinomial logistic regression was used for categorical variables, and ordered logistic regression was used for ordinal variables.

The within-group effects of the MBFPE program and the between-group effects of arm 1, and arm 2 were conducted using repeated measures analyses of variance (ANOVAs). Considering the differences at baseline, we controlled for the participating caregivers’ demographic information and the primary outcome variable (i.e., caregiver burden) in subsequent analyses. The effect sizes were calculated and explained according to Hedges, who provided benchmarks of Hedges’ g for small (*g* = 0.20), medium (*g* = 0.50), and large (*g* = 0.80) effects (65). SPSS version 23.0 and its computational tool PROCESS were used to perform all analyses.

## 3 Results

### 3.1 Summary of baseline information and comparison of participants

Sixty-five caregivers were included in the sample, including 33 caregivers assigned to the MBFPE program and the remaining 32 caregivers assigned to the FPE program in randomization. Among them, 51 caregivers (78.5%) were women and 42 (64.6%) were over 50 years old. In addition, 56 (86.2%) of the participating caregivers had obtained a secondary or tertiary degree, and 46 (71.8%) were married. Most of the caregivers (89.2%) still lived with the YAIR in their care, and more than 60% (43 caregivers) lived in a family of three to four members. However, 50 (76.9%) caregivers reported that they typically spent less than 10 hours per day providing care, with an average caregiving duration of 6.38 ± 6.04 hours. **Table 1** presents these statistics in detail and shows that there was no significant difference between arm 1 and arm 2 in terms of baseline information among the participating caregivers.

**Table 1:** Baseline information and comparison of caregivers (n=65).

Variables	MBFPE (n=33)	FPE (n=32)	*t*	*X^2^*	*p*
	n (%)	n (%)			
Gender Male Female	7 (21.2) 26 (78.8)	7 (21.9) 25 (78.1)		0.004	0.948
Age <40 40-50 51-60 >60	4 (12.1) 8 (24.2) 17 (51.5) 4 (12.1)	4 (12.5) 7 (21.9) 19 (59.4) 2 (6.3)	-0.197		0.844
Education Below Primary Primary Secondary Tertiary	2 (6.1) 4 (12.1) 17 (51.5) 10 (30.3)	0 3 (9.4) 15 (16.9) 14 (43.8)		2.920	0.404
Marriage Single Married Separated Widowed	5 (15.2) 21 (63.6) 6 (18.2) 1 (3.0)	4 (12.5) 25 (78.1) 2 (6.3) 1 (3.1)		2.444	0.485
Religion No Christianity Buddhism Other	23 (69.7) 6 (18.2) 3 (9.1) 1 (3.0)	20 (62.5) 10 (31.3) 2 (6.3) 0		2.394	0.495
Job Unemployed Searching Retired Part-time Full-time	7 (21.2) 7 (21.2) 5 (15.2) 4 (12.1) 10 (30.3)	4 (12.5) 7 (21.9) 8 (25.0) 1 (3.1) 12 (37.5)		3.945	0.557
Family income			-0.635		0.528
<20,000HKD	9 (27.3)	7 (21.9)			
20,000-49,999HKD	20 (60.6)	19 (59.4)			
50,000-99,999HKD	2 (6.06)	5 (15.6)			
>100,000HKD	2 (6.06)	1 (3.13)			
Live together Yes No	29 (87.9) 4 (12.1)	29 (90.6) 3 (9.4)		0.128	0.721
Number of Family Member 1-2 3-4 5-6	6 (18.2) 21 (63.6) 6 (18.2)	4 (12.5) 22 (68.8) 6 (18.8)	0.306		0.760
Hour of Caregiving <10 hours 10 – 20 hours >20 hours	26 (78.8) 5 (15.2) 2 (6.1)	24 (75.0) 6 (18.8) 2 (6.3)	0.803		0.425

We invited the caregivers to involve the YAIR in their care in the study. However, as participation in the study was voluntary, only 18 YAIR participants were included in the study. The comparison results showed that the basic demographic information and mental health conditions of the YAIR participants were not significantly different when their caregivers participated in the MBFPE or FPE program (see **Table 2**).

**Table 2:** Baseline information and comparison of YAIR (n=18).

Variables	MBFPE (n=8)	FPE (n=10)	*t*	*X^2^*	*p*
	n (%)	n (%)			
**Gender** Male Female	4 (50.0) 4 (50.0)	5 (50.0) 5 (50.0)		0.000	1.000
**Age** <20 20-30 >30	2 (25.0) 5 (62.5) 1 (12.5)	2 (20.0) 5 (50.0) 3 (30.0)	0.966		0.349
**Education** Secondary Tertiary	3 (37.5) 5 (62.5)	1 (10.0) 9 (90.0)		1.945	0.163
**Marriage** Single Married	8 (100.0) 0	9 (90.0) 1 (10.0)		0.847	0.357
**Religion** No Christianity	6 (75.0) 2 (25.0)	7 (70.0) 3 (30.0)		0.055	0.814
**Job** Unemployed/searching Part-time Full-time	7 (87.5) 1 (12.5) 0	7 (70.0) 0 3 (30.0)		3.825	0.281
**Diagnosis** Schizophrenia Psychosis	5 (62.5) 3 (37.5)	6 (60.0) 4 (40.0)		0.012	0.914
**Diagnosis Duration (month)** <12 12-24 >24	3 (37.5) 1 (12.5) 4 (50.0)	5 (50.0) 3 (30.0) 2 (20.0)	-1.701		0.108
**Family History of Psychiatric Disorders**				0.450	0.502
No	6 (75.0)	6 (60.0)			
Yes	2 (25.0)	4 (40.0)			

### 3.2 Within-group effects of the MBFPE program

For the Hypothesis 1, the within-group effects among the caregivers who participated in the MBFPE program were assessed to see how the caregivers in the MBFPE program changed across three time points. After controlling for their demographic information and caregiver burden, the results showed that caregiver burden (*g* = 0.208), caregiving experience of stigma (*g* = 0.323), effect on family (*g* = 0.282), physical distress (*g* = 0.303), depression (*g* = 0.371), anxiety (*g* = 0.429), well-being (*g* = 0.208), and interpersonal mindfulness, including compassion for children (*g* = 0.219) and listening with awareness (*g* = 0.235), had small effect sizes. Positive caregiving experiences had a medium effect size (*g* = 0.698), according to Hedges (66). Details can be found in **Table 3**.

**Table 3:** Group difference of MBFBE and FBE among pre-test, post-test, and follow-up for caregivers.

Variables	MBFBE (n=33)			FBE (n=32)			*Time* *F, p*, *g*	*Time X Group* *F, p*, *g*
	Pretest	Posttest	Follow-up	Pretest	Posttest	Follow-up		
ZBI	39.54 (13.95)	40.79 (15.52)	39.83 (13.52)	42.66 (17.51)	40.69 (15.47)	39.38 (18.40)	0.256, 0.721, 0.208	0.022, 0.883, 0.043
ECI (Stigma)	8.29 (4.47)	7.89 (4.62)	7.74 (4.16)	8.93 (3.84)	8.59 (4.11)	8.96 (4.53)	0.563, 0.574, 0.323	1.582, 0.215, 0.376
ECI (Effect on Family)	10.11 (6.06)	10.21 (6.20)	9.96 (4.91)	10.90 (5.25)	9.79 (4.86)	10.15 (5.62)	0.426, 0.656, 0.282	0.578, 0.451, 0.227
ECI (Positive Experience)	30.39 (7.13)	32.36 (7.63)	33.91 (6.72)	28.86 (6.47)	29.52 (7.74)	29.46 (7.93)	2.622, 0.085, 0.698	8.362, 0.006, 0.862
Physical Distress	26.79 (21.80)	27.79 (20.26)	31.83 (25.61)	38.21 (33.35)	30.45 (25.93)	33.88 (28.45)	0.484, 0.620, 0.303	0.764, 0.387, 0.260
HADS (Depression)	7.32 (3.75)	6.68 (3.79)	5.74 (4.01)	6.86 (4.69)	5.93 (4.64)	5.88 (5.19)	0.736, 0.485, 0.371	0.000, 0.982, 0.024
HADS (Anxiety)	7.57 (3.44)	7.11 (3.62)	7.09 (3.98)	8.83 (4.06)	7.48 (.87)	6.65 (4.28)	0.996, 0.378, 0.429	0.262, 0.612, 0.154
WHO-5	12.68 (5.70)	14.11 (5.03)	14.43 (3.70)	13.24 (5.38)	14.17 (4.06)	14.50 (5.56)	0.244, 0.785, 0.208	0.043, 0.838, 0.063
IMP (Total)	77.25 (12.41)	78.39 (9.51)	77.78 (6.67)	77.45 (10.01)	79.59 (10.37)	82.12 (11.19)	0.033, 0.926, 0.088	0.888, 0.351, 0.282
IMP (Compassion for Children)	26.29 (5.54)	27.00 (4.34)	27.09 (4.03)	27.03 (4.07)	26.45 (3.65)	28.08 (4.06)	0.939, 0.366, 0.419	0.389, 0.536, 0.188
IMP (Emotional Awareness in parenting)	17.50 (4.66)	16.89 (4.01)	16.65 (4.73)	17.21 (4.44)	18.17 (4.15)	18.38 (4.93)	0.015, 0.952, 0.063	0.987, 0.326, 0.296
IMP(Nonjudgmental Acceptance in Parenting)	19.96 (4.21)	20.75 (4.09)	20.52 (3.50)	20.52 (3.52)	20.79 (3.53)	21.73 (3.65)	0.058, 0.944, 0.108	0.071, 0.792, 0.088
IMP (Listening with Awareness)	13.50 (3.51)	13.75 (3.74)	13.52 (3.42)	12.69 (3.05)	14.17 (2.65)	13.92 (2.56)	0.295, 0.674, 0.235	0.053, 0.818, 0.063
NAS	31.75 (8.66)	31.32 (8.91)	32.09 (8.25)	33.07 (5.37)	33.07 (6.01)	35.69 (5.06)	0.236, 0.915, 0.088	0.142, 0.708, 0.108

ZBI, Zarit Burden Interview; ECI, Experience of Caregiving Inventory; HADS, Hospital Anxiety and Depression Scale; WHO-5, Five Well-Being Index; IMP, Interpersonal Mindfulness in Parenting Scale; NAS, Non-Attachment Scale.

For examining the effect sizes of the YAIR whose caregivers participated in the MBFPE program across three time points in the Hypothesis 2, their perceptions of caregiver EEs had a medium effect size (*g* = 0.499). Specifically, their perceived criticism (*g* = 0.458) and hostility (*g* = 0.218) had small effect sizes, while their perceived over-involvement (*g* = 1.263) had a large effect size. In addition, in the PANSS, their recovery level (*g* = 2.000), positive symptoms (*g* = 1.135), negative symptoms (*g* = 1.156), and general psychopathology (*g* = 0.976) had large effect sizes. In particular, their recovery level (p = 0.029) increased significantly and their levels of positive (p = 0.032) and negative symptoms (p = 0.029) decreased significantly from T1 to T3. The results are summarized in **Table 4**.

**Table 4:** Group difference of MBFBE and FBE among pre-test, post-test, and follow-up for patients.

Variables	MBFBE (n=8)			FBE (n=10)			*Time* *F, p*, *g*	*Time X Group* *F, p*, *g*
	Pretest	Posttest	Follow-up	Pretest	Posttest	Follow-up		
LEES	27.00 (10.07)	25.43 (12.10)	23.67 (11.04)	30.60 (9.32)	34.10 (10.43)	30.00 (6.72)	0.317, 0.735, 0.499	3.625, 0.086, 1.190
LEES_ Criticism	9.71 (3.90)	9.57 (4.54)	8.33 (4.03)	12.2 (2.30)	12.10 (3.41)	10.83 (3.55)	0.268, 0.643, 0.458	5.000, 0.049, 1.396
LEES_ Over-involvement	9.14 (3.19)	8.14 (4.41)	6.50 (2.95)	8.60 (3.53)	11.00 (3.56)	9.00 (3.23)	2.044, 0.209, 1.263	0.459, 0.514, 0.424
LEES_ Hostility	8.14 (3.89)	7.71 (4.19)	8.83 (4.36)	9.80 (4.47)	11.00 (4.40)	10.17 (3.31)	0.062, 0.827, 0.218	5.349, 0.043, 1.444
MHRM	109.14 (7.90)	119.57 (13.09)	113.50 (18.39)	112.40 (19.93)	113.00 (19.24)	113.67 (20.21)	5.120, 0.029, 2.000	0.136, 0.720, 0.227
PANSS _ Positive symptom	9.38 (2.13)	9.17 (2.64)	6.33 (3.33)	11.80 (5.31)	9.38 (2.50)	9.22 (2.59)	3.966, 0.032, 1.135	0.899, 0.363, 0.567
PANSS _ Negative symptom	12.38 (5.53)	9.00 (3.03)	7.83 (4.83)	14.90 (7.28)	12.63 (5.55)	13.44 (6.04)	4.100, 0.029, 1.156	1.937, 0.191, 0.830
PANSS _ General psychopathology	22.13 (4.42)	19.33 (3.27)	16.17 (8.38)	23.90 (8.33)	20.63 (4.31)	21.78 (5.26)	2.917, 0.073, 0.976	1.228, 0.291, 0.659

LEES, Level of Expressed Emotion Scale; MHRM, Mental Health Recovery Measure; PANSS, Positive and Negative Syndrome Scale.

### 3.3 Between-group analysis

**Figure 2:**
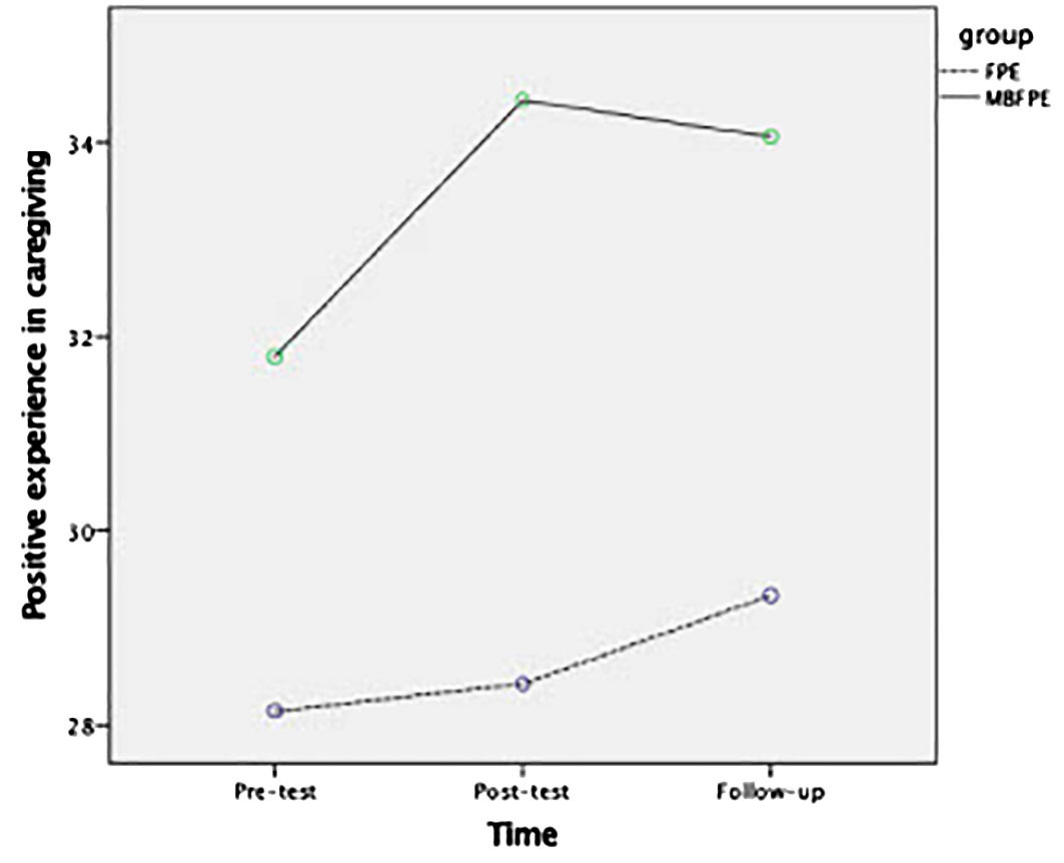
Estimated marginal means for caregivers' positive experience in MBFPE and FPE programs.

**Figure 3:**
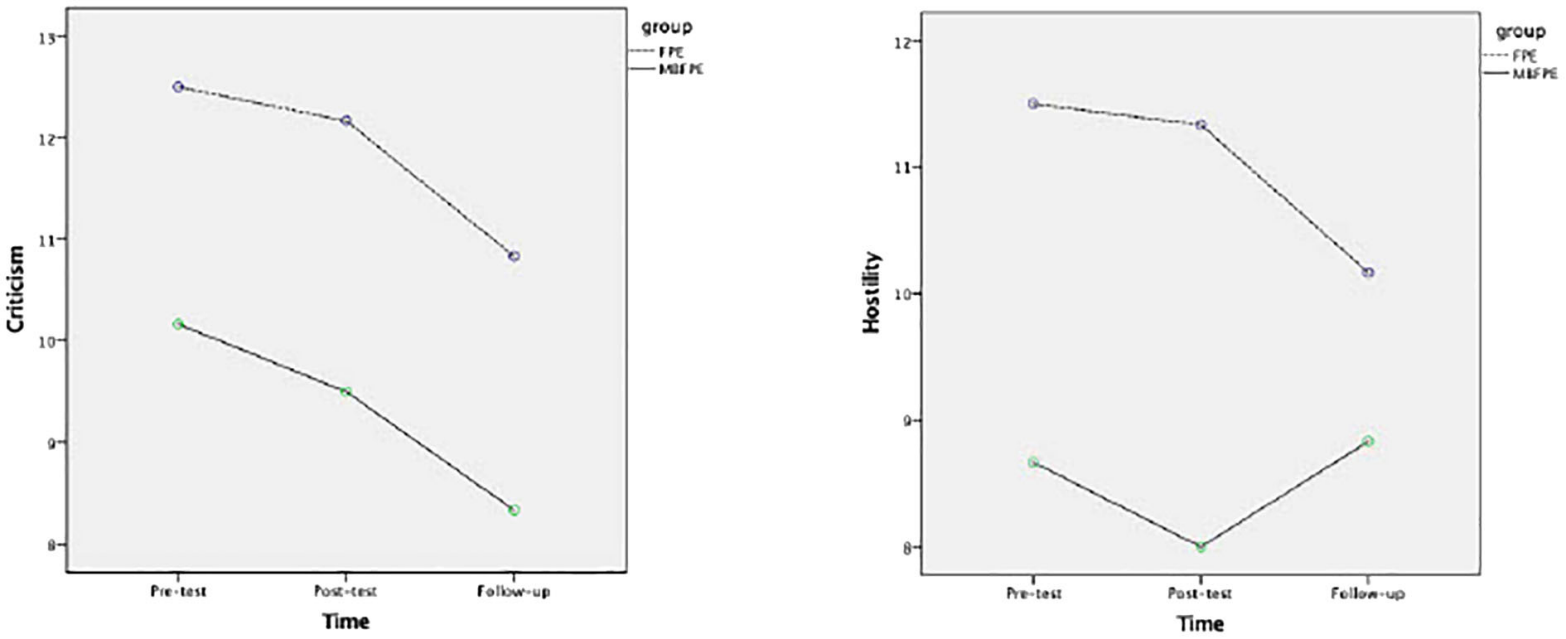
Estimated marginal means for criticism and hostility reported by YAIR in MBFPE and FPE programs.

Among the study variables for the participating caregivers, the Hypothesis 1 was further tested by examining the between-group effects to see the effects of MBFPE compared with the control group. The difference between the MBFPE and FPE programs was significant for positive caregiving experiences, which had a large effect size in the Time × Group analysis (*g* = 0.862, *p* = 0.006). No significant between-group differences were found for the other study variables. For the Hypotheses 2, among the YAIR participants, the difference between the MBFPE and FPE programs was significant for their perceptions of caregiver EEs, including large effect sizes of perceived criticism (*g* = 1.396, *p* = 0.049) and hostility (*g* = 1.444, *p* = 0.043). The results are reported in **Tables 3**, **4**, and **Figures 2**, **3** illustrate the estimated marginal means.

### 3.4 Moderation analysis of caregivers’ demographic information

**Figure 4:**
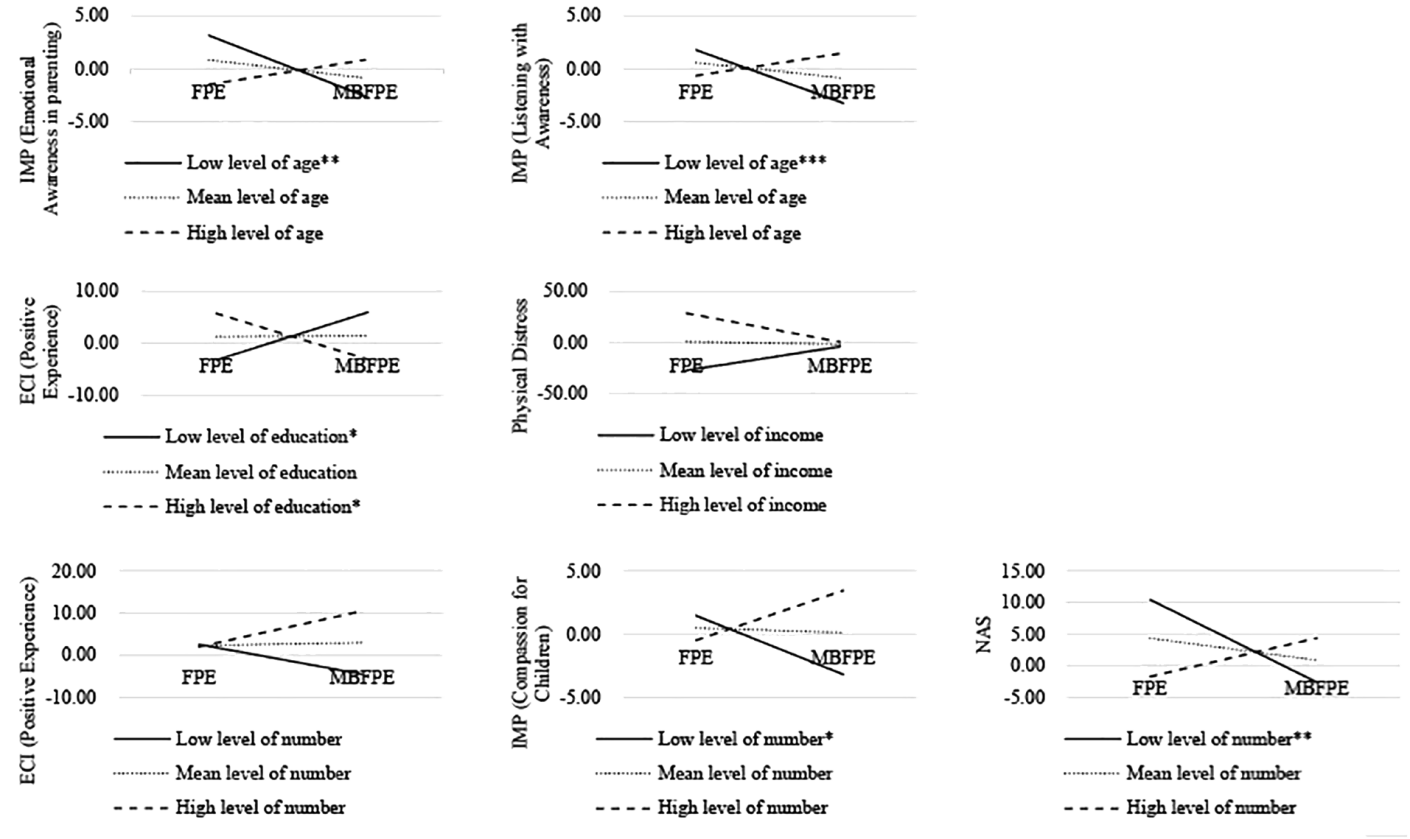
Significant moderating roles at low (1 standard deviation below the mean), mean, and high (1 standard deviation above the mean) levels of basic information among caregivers. *= *p*<0.05, **= *p*<0.01, ***= *p*<0.001.

After examining the within-group and between-group effect sizes, we further explored the potential moderating effects of the caregivers’ demographic information on outcomes in the study variables in the two arms for testing the Hypothesis 3. The significant moderating effects of the caregivers’ basic information are listed in **Table 5** and the interactions are illustrated in **Figure 4**.

**Table 5:** Significant moderators among basic information of caregivers.

Moderator	Variables	*b*	*SE*	95%*CI*	*t*	*p*	*R^2^*	*F*
Age	IMP (Emotional Awareness in parenting)	0.361	0.125	(0.105, 0.618)	2.898	0.008	0.593	2.798*
	IMP (Listening with Awareness)	0.320	0.080	(0.155, 0.485)	3.992	0.001	0.615	3.067**
Education	ECI (Positive Experience)	-12.502	3.959	(-20.656, -4.348)	-3.158	0.004	0.476	1.745
Income	Physical Distress	-0.001	0.000	(-0.001, -0.000)	-2.262	0.033	0.547	2.322*
Number of family member	ECI (Positive Experience)	6.084	2.588	(0.754, 11.414)	2.351	0.027	0.399	1.279
	IMP (Compassion for Children)	3.371	1.220	(0.860, 5.883)	2.764	0.011	0.464	1.667
	NAS	7.515	2.494	(2.378, 12.653)	3.013	0.006	0.472	1.720

*= *p*<0.05, **= *p*<0.01. SPSS Process Model 1 with 5000 bootstraps was adopted. The dependent variables and mediators are calculated as the change between follow-up and pre-test. The independent variable is group (0, FBE and 1, MBFBE). Non-significant moderation effects were not put in the table for saving space.

Compared with the caregivers in the FPE program, we observed fewer changes in emotional awareness in parenting and listening with awareness among the younger caregivers who participated in the MBFPE program (*t* = 2.898, *p* = 0.008). Those who were less educated (*t* = -3.158, *p* = 0.004) and lived with more family members (*t* = 2.351, *p* = 0.027) were more likely than other participants to gain positive caregiving experiences from participating in the MBFPE program.

In addition, those who lived with fewer family members reported significantly lower levels of changes in their compassion for children (*t* = 2.764, *p* = 0.011) and non-attachment (*t* = 3.013, *p* = 0.006). We also found that the caregivers with lower income reported more improvements in physical distress after participating in the MBFPE program (*t* = -2.262, *p* = 0.033). We also investigated the potential moderating effects of their marital status, job status, caregiving hours, and satisfaction with the program, and found no significant effects.

### 3.5 Fidelity and satisfaction with the program

Among the caregivers who participated in the MBFPE program, only 15 (45.4%) caregivers answered the question about the time spent on mindfulness exercises. The average time spent on mindfulness exercises was 10.3 ± 5.5 minutes per day. All the caregivers rated their satisfaction with the program in which they participated. Out of a score of 4, the results showed a mean score of 3.29 ± 0.41 for the MBFPE program and a mean score of 3.46 ± 0.34 for the FPE program. No significant difference in their satisfaction was found between the two groups (*t* = 1.793, *p* = 0.079).

In addition, an independent assessor with over 10 years of professional experience of conducting and teaching mindfulness-based training rated the MBFPE program using the MBI: TAC (61). The average score was 3.75 ± 0.81 (ranging from 3.0 to 5.0), showing good fidelity to the MBFPE program.

## 4 Discussion

Based on previous studies indicating the importance of MBPs for caregivers (66), we investigated the effects of an MBFPE program among Chinese caregivers of young adults with first-episode psychosis using an RCT comparing a 6-week MBFPE program with an ordinary FBP program. This study adds knowledge to the change mechanisms of the MBFPE program by exploring the role of EEs in the outcomes studied.

Hypothesis 1 was partially supported. Specifically, the results showed no significant differences in within-group effect sizes among the study variables for the caregivers who participated in the MBFPE program. Interestingly, however, we found a significant medium effect size on positive caregiving experiences in our between-group analysis. The caregivers who participated in the MBFPE program reported significant improvements in their positive experiences of parenting compared with those who participated in the FPE program at 9-month follow-up. After completing the MBFPE program, these participants may have found psychological resources in their caregiving process, although their caregiver burden did not show significant changes. These results are consistent with previous studies showing that the effect of a brief program on caregivers’ mental health is limited (24, 27). This finding is consistent with a recent trial in Japan, which showed that FPE had no positive effect on caregivers of people with recent onset psychosis (i.e., less than 5 years) (67).

Our results also confirmed our hypothesis that the MBFPE program could improve caregivers’ positive caregiving experiences. By participating in the MBFPE program, caregivers are more likely to make sense of their loved ones’ illness by accepting or allowing both positive and negative experiences, which can be called metacognitive capacity (68). The MBFPE program improves caregivers’ ability to form complex ideas about self and others and to integrate positive and painful events to be present. Studies have shown that such improvements can help caregivers cope with the functioning of their family members by focusing more on forming complex narratives and engaging in more meaningful interactions with a family member in recovery, leading to more positive caregiving experiences (18, 69).

Other study variables regarding caregivers were not significant when we examined their within-group and between-group effects. McFarlane also found no positive effects of FPE among caregivers of individuals with recent onset psychosis (24). However, our results could not confirm the results of systematic reviews (70, 71). These inconsistent results may have been due to several factors. First, caregivers who are still overwhelmed by their losses or life changes may find it difficult to learn and practice mindfulness exercises, as strong emotions may arise during periods of silence (29). Future studies could adjust the inclusion criteria to include caregivers whose family members began experiencing psychosis within the last 3 to 10 years, as caregivers may benefit more from mindfulness and a brief psychoeducation program when their family members with psychosis are relatively stable. Furthermore, the caregivers who participated in the FPE program showed slightly higher satisfaction with the program than those who participated in the MBFPE program. Both programs consisted of six 2-hour sessions. The caregivers in the FPE program were able to reflect on and discuss their psychological needs during each 2-hour session. However, those in the MBFPE program reported feeling restricted when discussing their emotional needs, as the mindfulness exercises took up half the time of each 2-hour session. The intensity of the MBFPE program may also have prevented the participating caregivers from thinking more about the intervention and gaining additional insights from it. In addition, the context of this study may have contributed to the non-significant changes. A high level of EE among families with a member suffering from psychosis was observed when Hong Kong and the world experienced the impact of COVID-19 with periods of lockdown and economic setback (13). At the time, most community mental health services had suspended their services, and such a brief program may not have provided adequate support for the extra burden placed on family members.

The results partially supported Hypothesis 2 related to YAIR. The recovery level of the YAIR participants whose caregivers participated in the MBFPE program increased significantly and their positive and negative symptoms decreased significantly between pre-test and 9-month follow-up, confirming the effectiveness of the MBFPE program in helping YAIR recover from their symptoms of psychosis. As expected, our results showed large effect sizes on EEs, including criticism and hostility, in the Time × Group analysis from T1 to T3. The YAIR participants whose caregivers participated in the MBFPE program reported significant decreases in criticism and hostility compared with those whose caregivers participated in the FPE program. The total EE and over-involvement scores over time revealed similar trends, so the non-significant between-group difference may be due to the small sample size.

This preliminary evidence indicates that MBFPE programs have a superior effect in reducing caregiver criticism, which has been suggested to be the most important component of EEs (12, 72). Caregivers’ criticism reflects their obvious disapproval or dislike of the YAIR in their care. Because of its measurable nature, perceived criticism may better reflect the effectiveness of MBFPE programs (12, 72). The MBFPE program in this study may have promoted caregivers’ acceptance and improved the conditions of the YAIR in their care, helping caregivers manage their emotions and expectations, and facilitating recovery and family functioning.

The improvements in EEs in this study also suggest that caregivers may benefit from emotion regulation by joining a program focused on mindfulness training. This finding also suggests that the different EE domains may be sensitive to cross-cultural variations (16, 17). Over-involvement and warmth in daily family interactions are indistinguishable in families influenced by Chinese and other collective cultures. With values supporting family unity, violation of adult children’s personal boundaries and caregivers’ over-involvement may be seen as signs of concern and care (73). MBFPE programs are likely to promote caregivers’ awareness and insight, but their effectiveness should be further verified in future studies with adequate sample sizes and across cultures.

The significant improvements in positive caregiving experiences and expressed criticism perceived by the YAIR participants at 9-month follow-up suggest that a brief program for caregivers has lasting effects. Once caregivers are equipped with mindfulness, the benefits may accumulate over time and become more evident at 9-month follow-up. Positive caregiving experiences can have a profound impact on family dynamics and support YAIR in their journey to recovery (18). More longitudinal studies of positive caregiving experiences, EEs, and YAIR recovery should be conducted to advance our practical knowledge of mental health care.

The caregivers’ demographic characteristics, including age, education level, family income, and number of family members, were found to moderate the effect sizes of the study variables, which partially supported Hypothesis 3. Older caregivers, less-educated caregivers, and those with a lower level of income benefited more from participating in the MBFPE program than other caregivers. One possible reason for this finding is that these populations responded more positively to the MBFPE program due to their limited opportunities to access mental health services (71). In contrast, the expectations and investments of younger caregivers with higher education and income levels may have prevented changes and improvements after the program, as they may have had more options and resources to cope with the burden of family care (71). Furthermore, in traditional Asian cultures, older adults are usually the heads of the household. As a result, it may be easier for them to reduce their caregiver burden after gaining awareness and insight, by recognizing the situations of the YAIR in their care (74, 75). Moreover, the caregivers living with more family members gained more positive caregiving experiences, had more compassion for the YAIR in their care, and greater non-attachment after participating in the MBFPE program. It may be easier for them to balance their focus on other family members and be less fixated on the fluctuating conditions of the YAIR in their care.

This study has some limitations that should be acknowledged. First, our analyses were based on a small sample of caregivers due to the difficulty of recruiting participants during the COVID-19 pandemic. Some of the caregivers expressed health concerns and explicitly indicated that they would prefer an online program, even if they were interested in our program. It is uncertain whether such positive results can be generalized to other contexts.

Second, of the 65 caregivers who participated in the study, only 27.7% of the YAIR in their care contributed to this research; as such, recruiting YAIR participants was much more challenging than expected. Our research team made great efforts to promote the study in outpatient clinics, but in-depth conversations with potential participants were discouraged due to the COVID-19 pandemic. Some of the caregivers preferred not to let their family members know that they had joined this study, to avoid unexpected reactions from the YAIR in their care. Further studies could recruit participants using different strategies, such as using medical records after receiving informed consent from YAIR.

Finally, we investigated EEs based on the perceptions of the YAIR participants rather than objective measures, such as observing interactions between caregivers and YAIR. We also did not collect data from caregivers for their EEs, so the results for EEs may suffer from reporting bias, as YAIR’s improved mental health may have affected their perceptions of caregiver EEs. Future studies should include more measures of EEs in multiple dimensions, such as by using 5-minute speech samples, a well-established behavioral coding to measure EEs based on caregiver behavior (76). It would also be useful to adopt a more rigorous study design and explore the mediating effects of EEs on family program outcomes.

## 5 Conclusions

This study provides evidence of the benefits of MBPs for caregivers of young adults with first-episode psychosis. The results showed that changes in caregivers have spillover effects by promoting the recovery of their care recipients. The role of EEs in these encouraging results should be further explored in future studies. The application of mindfulness training to promote informal family care for people recovering from psychosis should be encouraged.

## Data Availability

The raw data supporting the conclusions of this article will be made available by the authors, without undue reservation.
