# Supplementary material for: Mindfulness-based family psychoeducation intervention for caregivers of young adults with first-episode psychosis: results at 9-month follow-up
Source: Front Psychiatry. 2024 Sep 26;15:1460151. doi: 10.3389/fpsyt.2024.1460151 (PMC11464292; doi:10.3389/fpsyt.2024.1460151)
Supplement: Supplementary file 1 [file DataSheet1.pdf]

Appendix 1

Brief outline of mindfulness-based family psychoeducation (MBFPE) (arm 1) and family psychoeducation (FPE) (arm 2).

Session themes	Mindfulness-based family psychoeducation (MBFBE) (arm 1)	Family psychoeducation (FPE) (arm 2)
(1) Understanding the impact of caregiving stress	(a) Orientation to the program<br>(b) Mindfulness practice: mindful eating, body scan<br>(c) Video: caregiver’s reaction of onset of SMI<br>(d) Discussion: awareness of the impact of caregiving on body and mind<br>(e) Homework: body scan	(a) Orientation to the program<br>(b) Sharing and discussion: stress and reactivity in caregiving<br>(c) Video: caregiver’s reaction of onset of SMI<br>(d) Discussion: normalizing the reactions of caregiver stress
(2) The impact of psychosis to young psychosis	(a) Mindfulness exercises: mindful stretching, mindful walking<br>(b) Inquiry: mindfulness exercises<br>(c) Video show: understanding positive and negative symptoms<br>(d) Homework: mindful stretching, 3 min breathing, and photovoice	(a) Sharing and discussion: issues in handling symptoms and behaviors of family member in recovery<br>(b) Video show: understanding positive and negative symptoms<br>(c) Discussion: strategies on symptom management and promoting recovery
(3) The experience of young adults with psychosis in recovery	(a) Mindfulness exercises: mindful sitting, mindful communication<br>(b) Inquiry: mindfulness exercises and photovoice<br>(c) Video show: sharing of persons in recovery<br>(d) Homework: mindful sitting, 3 min breathing, and photovoice (an unpleasant moment)	(a) Sharing and discussion: goals and needs for holistic recovery<br>(b) Video show: sharing of persons in recovery<br>(c) Discussion on understanding and communicating with family members in recovery
(4) The struggles of caregivers	(a) Mindfulness exercises: mindfulness with difficult moments, mindful communication<br>(b) Inquiry: mindfulness exercises and photovoice<br>(c) Video show: challenges in caregiving and self- care<br>(d) Homework: mindfulness with difficult moments, 3-min breathing, and photovoice (my family)	(a) Sharing and discussion: stress and coping in caregiving, and difficulties in communicating with family members in recovery<br>(b) Video show: challenges in caregiving and self-care<br>(c) Discussion on preventing compassion fatigue
(5) partnership with multi-disciplinary team in recovery	(a) Mindfulness exercise: be-friending<br>(b) Inquiry: mindfulness exercise and photovoice<br>(c) Video show: understanding treatment and services fo people in recovery<br>(d) Homework: be-friending, 3 min breathing, and photovoice (what I learned in this course)	(a) Sharing and discussion: experiences and issues about working with mental health professionals<br>(b) Video show: understanding treatment and services for people in recovery<br>(c) Discussion on strategies for promoting recovery and partnership with professionals
(6) Review of learning	(a) Mindfulness exercises: body scan, mindful sitting<br>(b) Inquiry: mindfulness exercises and photovoice<br>(c) Video show: relapse plan and management<br>(d) Review: what I learn in the program	(a) Sharing and discussion: risk and relapse management<br>(b) Video show: relapse plan and management<br>(c) Review: what I learn in the program
